# ﻿*Ixeridiumsagittarioides* (Asteraceae-Cichorieae) revisited: range extension and molecular evidence for its systematic position in the *Lactuca* alliance

**DOI:** 10.3897/phytokeys.230.107733

**Published:** 2023-08-07

**Authors:** Jian-Wen Zhang, Norbert Kilian, Jiang-Hua Huang, Hang Sun

**Affiliations:** 1 CAS Key Laboratory for Plant Diversity and Biogeography of East Asia, Kunming Institute of Botany, Chinese Academy of Sciences, Kunming 650201, Yunnan, China CAS Key Laboratory for Plant Diversity and Biogeography of East Asia, Kunming Institute of Botany, Chinese Academy of Sciences Kunming China; 2 Botanischer Garten und Botanisches Museum Berlin, Freie Universität Berlin, Königin -Luise-Str. 6–8, 14195 Berlin, Germany Freie Universitat Berlin Berlin Germany; 3 Forestry Bureau of Wangmo County, Wangmo 552300, Guizhou, China Forestry Bureau of Wangmo County Wangmo China

**Keywords:** Asteraceae, Cichorieae, Crepidinae, Lactucinae, *Lactucasagittarioides*, reticulate evolution, systematic position, taxonomy

## Abstract

Our first record of the rare and scatteredly distributed *Ixeridiumsagittarioides* for Guizhou, China, triggered a study to assess its systematic position. The species was placed in four different genera in the course of its taxonomic history and was recently treated with doubts as a member of *Ixeridium* in the Flora of China. Comparative morphological investigation and phylogenetic analyses based on the nuclear ribosomal DNA internal transcribed spacer (nrITS) and five non-coding plastid DNA regions (*petD* region, *psbA-trnH*, *trnL-trnF*, *rpl32-trnL* (UAG) and *5´rps16-trnQ* (UUG) spacers) provided evidence that the species is not a member of *Ixeridium* and the Crepidinae but has evolved by ancient hybridisation of members of the *Lactuca* alliance (Lactucinae). It is reinstated as *Lactucasagittarioides* and a comprehensive morphological description is provided, based on material from its entire range of distribution.

## ﻿Introduction

**Figure 1.**
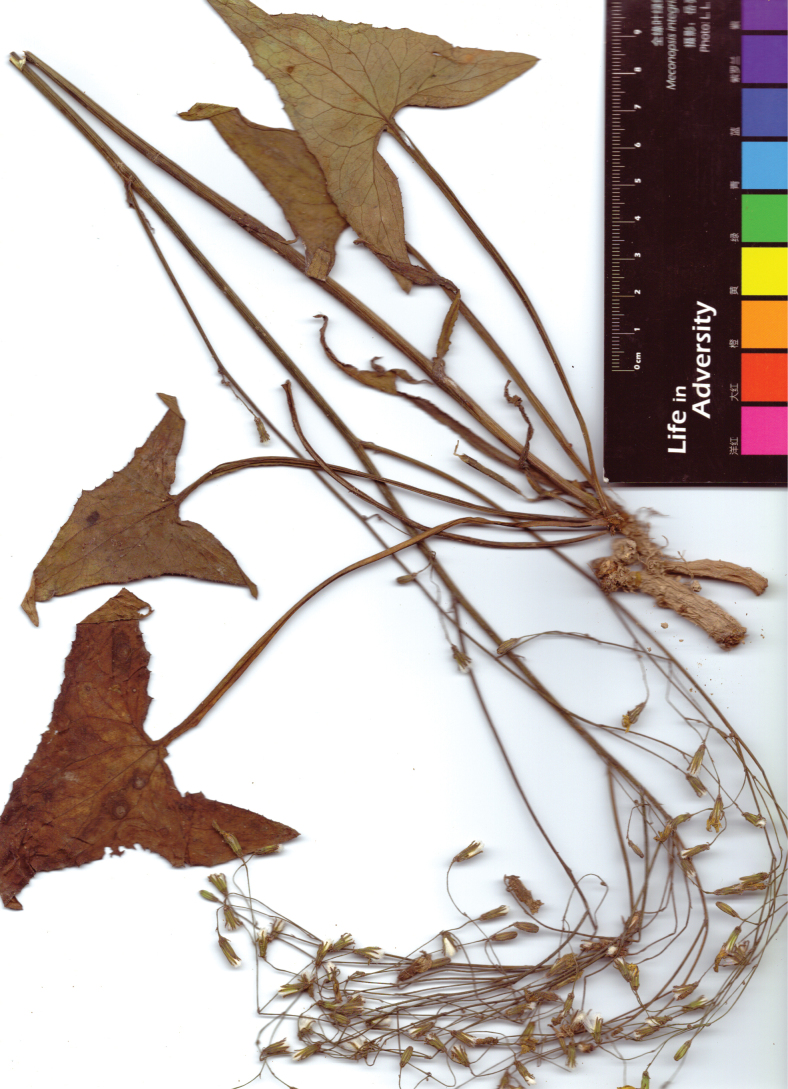
*Lactucasagittarioides* – specimen collected in Guizhou at Wangmo, 25.18°N, 106.12°E, 13 Apr 2018, *J.W. Zhang 1091* (KUN).

A perennial herb with very conspicuous, usually long-petiolate sagittiform rosette leaves and a scattered distribution along the Himalayan mountain chain from N Pakistan and NW India across Nepal, Bhutan, N Myanmar and N Thailand to Yunnan (China), was originally described as *Lactucasagittarioides* C.B.Clarke (Clarke 1876) based on material from NW India, Nepal and Burma (Shi and Kilian in Shi et al. 2011). Later, Stebbins (1937a) removed it from *Lactuca* and placed it in *Ixeris* as *I.sagittarioides* (C.B.Clarke) Stebbins, Pak and Kawano (1992) moved it then to *Ixeridium* as *I.sagittarioides* (C.B.Clarke) Pak & Kawano, whereas Sennikov (1997) placed it in *Mycelis* as *M.sagittarioides* (C.B.Clarke) Sennikov. Today, we know from molecular phylogenetic analyses that *Lactuca* and *Mycelis* are members of the subtribe Lactucinae, while *Ixeris* and *Ixeridium* are a sister group in the subtribe Crepidinae (Kilian et al. 2009a, 2017; Wang et al. 2013; Nakamura et al. 2014; Wang et al. 2020; Güzel et al. 2021). Both subtribes were only recently separated (Bremer 1994), lack exclusive morphological synapomorphies (Bremer 1994; Kilian et al. 2017), and disentangling them even resulted in the splitting of genera (e.g. Zhang et al. 2011a, b). Shi and Kilian (in Shi et al. 2011) expressed doubts at the placement of the species in *Ixeridium* and the Crepidinae but left the problem unsolved and up for further studies. However, *Ixeridiumsagittarioides* so far has not been included in any phylogenetic study and its systematic position has not been addressed. A first record of the species from SW Guizhou made by us in 2018 (Fig. 1) then triggered a study of *I.sagittarioides*, and this contribution has the aim to reconsider its systematic position based on morphological and molecular phylogenetic investigations.

## ﻿Materials and methods

### ﻿Plant material

The study was based on the gathering of herbarium and tissue material for DNA isolation of *Ixeridiumsagittarioides* from Guizhou, deposited in KUN, additional herbarium samples of this species from the herbaria of E, IMDY, K, KUN and PE, and further herbarium material of other species for morphological comparison from the herbaria of B, KUN, M and MSB (herbarium codes according to Index Herbariorum, http://sweetgum.nybg.org/science/ih/). In addition, digital images of specimens at BM and L were consulted through GBIF (https://www.gbif.org/species/3100771). To avoid wrong conclusions due to misidentification, occurrence records not substantiated by physical or digital specimens were not taken into account.

### ﻿DNA extraction, amplification and sequencing

Extraction of DNA and amplification of markers for the accession of *Ixeridiumsagittarioides* followed the protocols by Wang et al. (2013) and, as in that study, the nrITS region and five non-coding plastid DNA markers, the *petD* region and the spacers *psbA-trnH*, *trnL-trnF*, *rpl32-trnL* (UAG), *5*’*rps16-trnQ* (UUG), were used. PCR products were purified with a QIAquick PCR Purification Kit (BioTeke, Beijing, China) and sequenced using an ABI 3730XL automated DNA sequencer (Applied Bio-systems, Foster City, California, U.S.A.). The sequences were deposited through GenBank (Table 1).

**Table 1.** INSDC (International Nucleotide Sequence Database Collaboration) accession numbers of newly generated nrITS and plastid DNA sequences with specimen data of the sample used.

Sample	Specimen	Locality	Date	Marker: accession no.
*Lactuca sagittarioides_ ZJW1091*	*J. W. Zhang 1091* (KUN)	China, Guizhou, Wangmo, 25.18°N, 106.12°E, 700 m	13 Apr 2018	nrITS: OR196839; *petD*: OR221191; *psbA-trnH*: OR221192; *trnL-trnF*: OR221190; *rpl32-trnL* (*UAG*): OR221193; *5‘rps16-trnQ* (*UUG*): OR221194.

### ﻿Sampling and sequence alignment

The *Ixeridiumsagittarioides* sequences were initially included in the separate nrITS and plastid DNA matrices built by Kilian et al. (2017), aligned with MAFFT v.7 using default parameters (Katoh et al. 2017) and adjusted manually using PhyDE v.0.9971 (Müller et al. 2010). Indels were coded as binary characters using simple indel coding (Simmons and Ochoterena 2000) implemented in SeqState v.1.40 (Müller 2005); inversions were re-inverted. The nrITS matrix was subdivided into the four partitions ITS1, 5.8S, ITS2, indels. The plastid DNA matrix was subdivided into six partitions, one for each of the markers and a binary partition for the coded indels. Length-variable mononucleotide portions and hypervariable sections were excluded because of homology uncertainty. After an initial tree calculation with MP (see below) to infer the subtribal systematic position of *I.sagittarioides* based on either matrix, the sampling in the two original matrices was strongly condensed with a focus on the next related taxa in the initial reconstruction, and the nrITS matrix was supplemented by an accession of *Lactucaadenophora* from Güzel et al. (2021). Voucher data and INSDC (International Nucleotide Sequence Database Collaboration, including GenBank/EMBL/DDBJ) accession numbers of the published sequences are given in Kilian et al. (2017: appendix 1) and Güzel et al. (2021: Online Resource 1).

### ﻿Phylogenetic reconstructions

Phylogenetic relationships were inferred using maximum parsimony (MP), maximum likelihood (ML) and Bayesian inference (BI). The last two were run on the high-performance computing system of the Freie Universität Berlin (Bennett et al. 2020). MP was performed with the parsimony ratchet using PRAP v.2.0 (Müller 2004) with 10 additional random cycles and default parameters in combination with PAUP v.4.0b10 (Swofford 2003); Jackknife (JK) support values were calculated in PAUP with 10,000 replicates using the TBR branch swapping algorithm with 36.788% of characters deleted and one tree held during each replicate. ML analyses were done with the MPI version of RAxML-NG 0.9.0 (Kozlov et al. 2019). The best-fit evolutionary models were searched with ModelTest-NG (Darriba et al. 2019) and selected according to the Bayesian Information Criterion: SYM+G4 for ITS1, TrNef+I+G4 for 5.8S, TIM3ef+G4 for ITS2; TPM1uf+G4 for the *petD*-region and the *trnL-F* spacer and TVM+G4 for the *psbA-trnH*, *trnQ-rps16* and *rpl32-trnL* spacers; the binary indel partitions were not included due to software restrictions. The tree space was explored with 50 tree searches using 25 random and 25 parsimony-based starting trees. Standard bootstrapping was done employing the bootstopping test with a bootstrap convergence requirement of 3% default cut-off; the support values were mapped onto the best-scoring tree. The BI analyses were performed with the MPI version of MrBayes (Ronquist et al. 2012). The best-fit evolutionary models were sampled across the general time reversible (GTR) model space in the Bayesian MCMC analysis (Huelsenbeck et al. 2004) and two simultaneous runs of four parallel chains each were performed for 3 × 10^7^ generations with a sample frequency of 1 tree per 2000 generations and a conservative burn-in of 20%. Convergence of the runs was ensured by having the post-burn-in average standard deviation of split frequencies below 0.01 and an effective sampling size (ESS) of some 1000s in either run for all parameters. TreeGraph v.2 (Stöver and Müller 2010) was used to visualize the trees with statistical node support.

## ﻿Results

### ﻿Phylogenetic analysis

**Figure 2.**
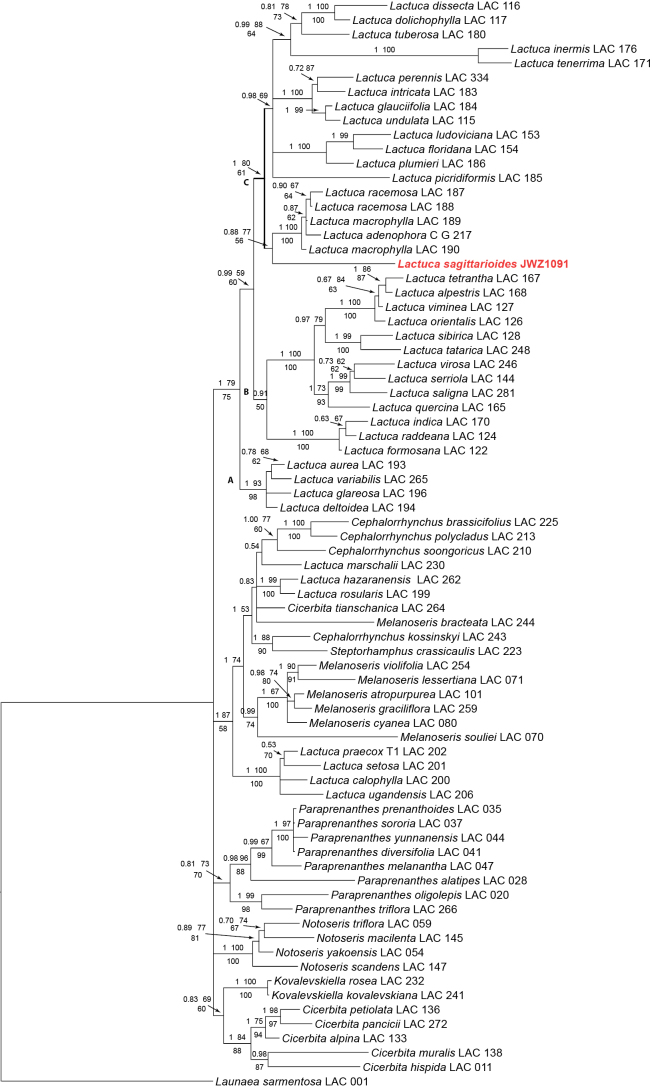
Majority consensus phylogram of the Lactucinae from Bayesian analysis (support values: first line Bayesian posterior probability / maximum likelihood bootstrap; second line maximum parsimony jackknife) based on the nrITS region.

The aligned nrITS region of 76 samples had a length of 687 characters; together with the coded indels the matrix included a total of 778 characters, of which 298 were parsimony-informative. The MP analysis resulted in 1532 most parsimonious trees (L = 1391, CI = 0.479, RI = 0.674, RC = 0.323, HI = 0.521), largely congruent in topology with the trees of the BI and ML analyses. Fig. 2 shows the BI majority consensus phylogram with the BI posterior probabilities (PP) and ML bootstrap (BS) support values (bootstrapping converged after 1400 replicates) below the branches and the MP jackknife (JK) support values above the branches.

**Figure 3.**
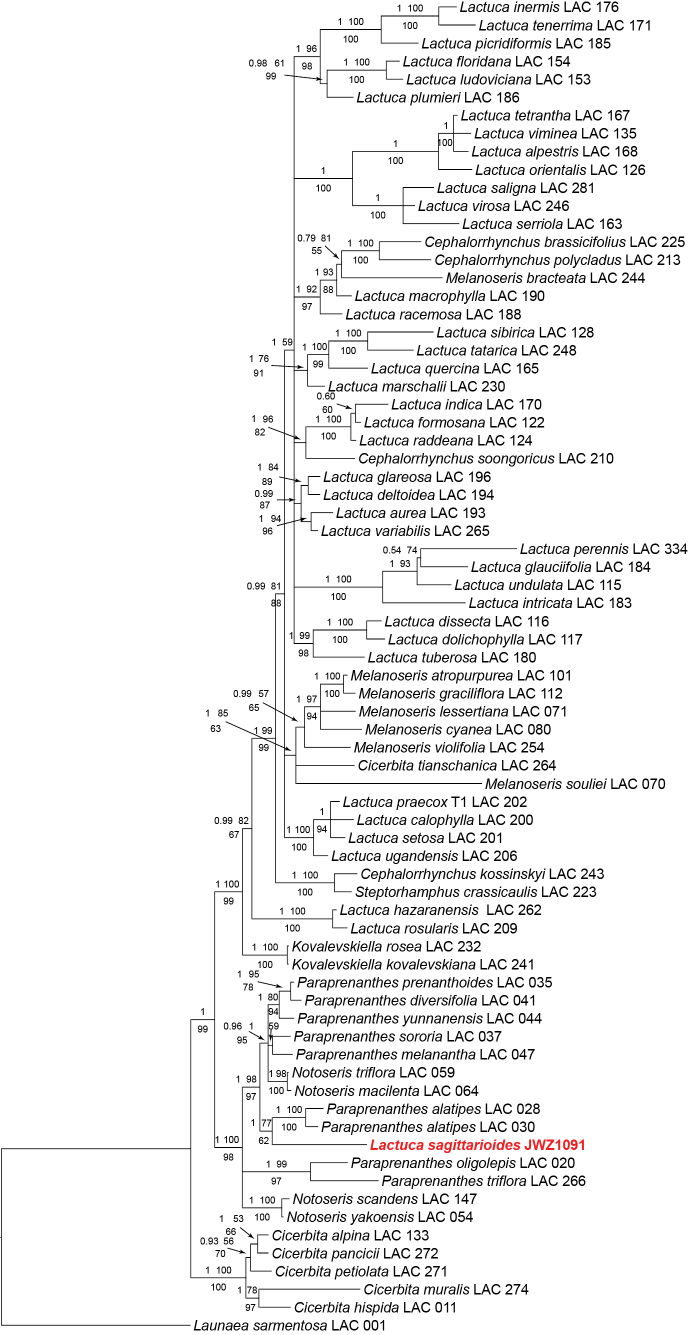
Majority consensus phylogram of the Lactucinae from Bayesian analysis (support values: first line Bayesian posterior probability / maximum likelihood bootstrap; second line maximum parsimony jackknife) based on the five non-coding plastid DNA regions.

The final aligned concatenated plastid DNA markers of 74 samples had a length of 5827 characters; together with the coded indels the matrix included a total of 6207 characters, of which 504 were parsimony-informative. The MP analysis resulted in 1720 most parsimonious trees (L = 1627, CI = 0.770, RI = 0.814, RC = 0.627, HI = 0.230), largely congruent in topology with the trees of the BI and ML analyses. Fig. 3 shows the BI majority consensus phylogram with the BI posterior probabilities (PP) and ML bootstrap (BS) support values (bootstrapping converged after 300 replicates below the branches and the MP jackknife (JK) support values above the branches.

*Ixeridiumsagittarioides* is deeply nested in the subtribe Lactucinae both in the nrITS (Fig. 2) and the plastid DNA phylogeny (Fig. 3), but at different positions. In the nrITS phylogeny, the species is nested in the *Lactuca* clade (PP 1, BS 79, JK 75) and resolved as sister to the strongly supported *Lactucaracemosa*-*L.macrophylla* clade with very weak support (PP 0.88, JK 56) in the Bayesian and MP analyses, and only moderate support (BS 77) in the ML analysis. In contrast, in the plastid DNA phylogeny, the species is nested in the earlier diverging *Notoseris-Paraprenanthes* clade and resolved as sister to *Paraprenanthesalatipes* (Collett & Hemsl.) Z.Wei & S.X.Zhu = *Lactucaparishii* Craib with strong support (PP 1) in the Bayesian, moderate support (BS 77) in the ML and weak support (JK 62) in the MP analysis.

### ﻿Morphology

**Figure 4.**
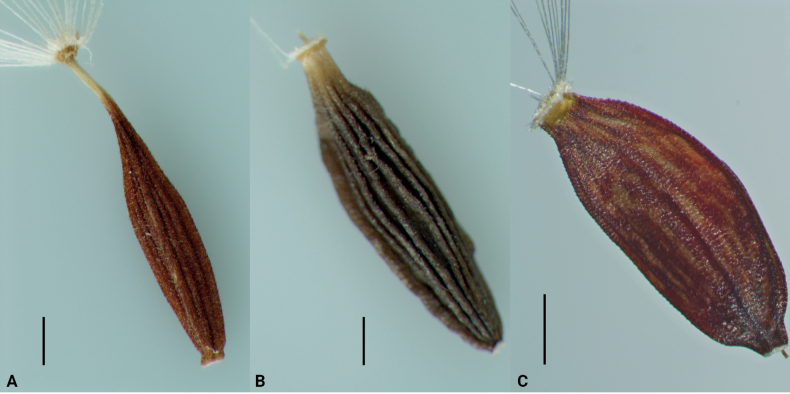
Achenes **A***Lactucasagittarioides*; specimen: China, Yunnan, Forrest 29519 (E 00489230) **B***Paraprenanthestriflora*; specimen: Nepal, Pokahara region, between Tikhedhunga and Ghorepani. 19 Sep 2008, A. Suchorukow N-15 (B) **C***Lactucamacrophylla*; specimen: Turkey Artvin, Şavşat, Pınarlı Köyü, Balıkgölü girişi, kalıntı bitkiler arası, 2035 m, 13 Sep 2014, Coşkunçelebi & Güzel 339 (KTUB). Scale bars: 0.5 mm (**A, B**); 1 mm (**C**); photographs by Murat Güzel.

For the comparison of *Lactucasagittarioides* with *Ixeridium* on the one hand and its sister clades in the subtribe Lactucinae inferred from the molecular phylogenetic analyses of the nrITS and the plastid DNA matrices on the other hand, diagnostic morphological characters, in particular of the achenes (Fig. 4), were used. The results are summarised in Table 2 and show that, in contrast to superficial resemblance through capitulum shape and corolla colour, the species also differs from *Ixeridium* in achenes morphology. Congruence is highest with *Paraprenanthes*, apart from the different capitulum shape and corolla colour, whereas also achene and pappus morphology of the *Lactucaracemosa*-*L.macrophylla* clade does not well match with that of *L.sagittarioides*.

**Table 2.** Diagnostic morphological features of *Lactucasagittarioides* in comparison with the genus *Ixeridium* and the related Lactucinae members inferred from the molecular phylogenetic analyses of the nrITS and the plastid DNA matrices, respectively.

Diagnostic features	*Lactucasagittarioides*	*Ixeridium*	*Lactucaracemosa*-*L.macrophylla* clade	*Paraprenanthes*
Capitula	moderately narrowly cylindrical	moderately narrowly cylindrical	moderately narrowly to broadly cylindrical	narrowly cylindrical
Corolla, colour	yellow	yellow	cyanic	cyanic
Achenes, ribbing pattern	5 main ribs each accompanied by 2 secondary ribs; lateral ribs not enlarged	10 equal ribs, none winged	usually 4 main ribs each accompanied by 2 secondary ribs, lateral ribs winglike enlarged	5 main ribs each accompanied by 2 secondary ribs; lateral ribs not enlarged
Achenes, ornamentation	muricate	± smooth	faintly muricate	muricate
Achenes, carpopodium	callose, uninterrupted annular	± callose, uninterrupted tubular	callose, uninterrupted annular	callose, uninterrupted annular
Pappus, composition	one series of bristles	one series of bristles	one series of bristles and outer row of minute hairs	one series of bristles
Pappus, colour	white	yellowish or straw-coloured	white	white

## ﻿Discussion

### ﻿Systematic position of *Ixeridiumsagittarioides*

Molecular phylogenetics

Our molecular phylogenetic analyses resolved *Ixeridiumsagittarioides* unanimously as a member of subtribe Lactucinae and therefore deprive the basis for the placement in *Ixeridium* and the Crepidinae. Otherwise, the analyses revealed a surprising cytonuclear discordance (Lee-Yaw et al. 2018). All three phylogenetic reconstructions based on the nrITS matrix placed the species deeply in the *Lactuca* clade. There, it is resolved in a sister group relationship to the *L.racemosa*-*L.macrophylla* clade, but with very weak (BI and MP) or moderate (ML) support and at a comparatively long branch, indicating genetic distance (Fig. 2). Hence whereas the placement in *Lactuca* must be considered well supported, the indicated relationship to the *L.racemosa*-*L.macrophylla* clade should be taken with caution, in view of the dissimilarities in achene morphology, and may be even suspected as a result of long branch attraction (Bergsten 2005). The plastid DNA analyses, resolved the species in a very well supported clade including the E Asian genera *Notoseris* and *Paraprenanthes*. This clade is known from the previous analyses based on plastid DNA by Wang et al. (2013) and Kilian et al. (2017). Within the *Notoseris-Paraprenanthes* clade, the species forms a sister group relationship with *Paraprenanthesalatipes*. However, the altogether moderate statistical support and the comparatively long branch (Fig. 3) speak against a very close relationship.

#### ﻿Morphology

The achenes of *Lactucasagittarioides* (Fig. 4A) are slightly compressed and have 5 main ribs accompanied by usually 2 secondary ribs (best seen in the middle third), thus altogether usually have 15 ribs, sometimes a single secondary rib may be (partly) missing. Of the five main ribs two are in lateral position, one in the middle of the adaxial face and two are about equally spaced on the abaxial face. This ribbing pattern is considered plesiomorphic for the tribe (Kilian et al. 2009a) and is also present in both the Lactucinae and Crepidinae. In the Lactucinae, reductions in the number of main ribs as well as of the number of secondary ribs are frequent (Stebbins 1937b, 1940; Kilian et al. 2009a). In the Crepidinae, in contrast, both increases (Stebbins 1940) and reductions in the number of the secondary ribs have evolved, whereas the number of the main ribs is assumed to have been unchanged. Shape and prominence of main and secondary ribs have become equal in several groups. Achenes with only 10 ribs, by fusion of the adjacent secondary ribs, characterise *Askellia* (Pak 1993 sub Crepissect.Ixeridopsis), *Ixeris* and *Ixeridium* (Pak and Kawano 1990 sub *Ixeris* s.l.), and are a synapomorphy of the *Askellia-Ixeris-Ixeridium* clade (see the phylogenetic backbone of the Crepidinae based on nrITS by Wang et al. 2020). Apparently, the ribbing pattern of the achenes of *L.sagittarioides* is clearly different from that of *Ixeridium*. The achenes of *L.sagittarioides*, moreover, have a callose non-interrupted annular carpopodium (Haque and Godward 1984), which is characteristic for members of the subtribe Lactucinae (Kilian et al. 2009a), whereas in the Crepidinae interrupted callose carpopodia predominate. *Ixeris* and *Ixeridium*, however, are exceptions and have uninterrupted, somewhat tubular carpopodia, which may have contributed to their former placement along with *Lactuca* by Bentham (in Bentham and Hooker 1873). Hence, the systematically relevant achene morphology provides further evidence against a placement of *L.sagittarioides* in *Ixeridium* and the Crepidinae, and corroborates its placement in the Lactucinae. Also with respect to pappus colour, *L.sagittarioides* disagrees with *Ixeridium*, because all species clearly known to belong to *Ixeridium* have a straw-coloured or yellowish pappus, a feature which also distinguishes the genus from its sister group *Ixeris*.

Within the Lactucinae, the weakly supported sister group relationship with the *Lactucaracemosa*-*L.macrophylla* clade in the nrITS phylogeny does not agree well with achene morphology (Fig. 4C): the members of this clade are characterised by distinctly compressed achenes with four main ribs, narrowly winged lateral ribs, usually rather weak or inconspicuous secondary ribs, a faintly muricate surface and a pappus with an outer row of minute hairs; hence markedly different to the achenes of *L.sagittarioides*. The yellow flower colour of *L.sagittarioides* also disagrees with the cyanic flower colour of the three members of that clade. Achenes of the plesiomorphic constitution with five main ribs, well developed secondary ribs and a less strongly compressed corpus are, however, still present in most other terminal clades of *Lactuca*, including the larger clade C (Fig. 2), to which the *Lactucaracemosa*-*L.macrophylla* clade belongs. This also holds for the yellow flower colour, which, moreover, is not only known to vary within clades but sometimes also within a species, examples being *L.tuberosa* Jacq. and *L.inermis* Forssk.

The sister group relationship with *Paraprenanthesalatipes* of the *Notoseris-Paraprenanthes* clade revealed in the plastid DNA marker phylogeny, in contrast, shows a better agreement with achene morphology: shape, ribbing pattern, surface ornamentation and also pappus structure of *L.sagittarioides* and *Paraprenanthes* principally match (Fig. 4A, B). However, in view of this more widespread, plesiomorphic constitution, the resemblance with *L.sagittarioides* is not very conclusive for a closer phylogenetic relationship. Other morphological features, such as the exclusively cyanic or purple flower colour and predominantly few-flowered, slender capitula in the *Notoseris-Paraprenanthes* clade even speak against a very close relationship.

*Lactucasagittarioides* is distributed along the lower escarpments of the Himalaya belt and extends into the mountain ranges adjacent to the east in Yunnan and Guizhou. The new record from central northern Guizhou makes its presence also in the province of Sichuan rather likely. The *Lactuca* lineage (in the sense of Kilian et al. 2017 and Jones et al. 2018) is distributed chiefly from the Mediterranean Basin and Europe along the Alpine-Himalayan belt (Storetvedt 1990) across SW to E Asia, and also in North America. Its region of origin is the E Mediterranean-SW Asian area according to Kilian et al. (2017), where also the *Lactucaracemosa*-*L.macrophylla* clade is present, being restricted to the Caucasus, Ural Mts., Turkey and N Iran (Güzel et al. 2021). *Paraprenanthesalatipes*, in contrast, is distributed in a rather small area at the easternmost edge of the Alpine-Himalayan belt in SW Yunnan, N Myanmar, N Thailand and N Vietnam. A plausible scenario according to the molecular phylogenetic findings would be that *Lactucasagittarioides* originates from the hybridisation of a *Lactuca* ancestor on its eastward migration from SW Asia along the Alpine-Himalayan belt with a *Paraprenanthes* ancestor extending its area of distribution westwards.

The context of the subtribe lends further support to such a scenario when we consider the numerous reticulation events at various depths of the species tree to be concluded from previous studies (Liu et al. 2013; Wang et al. 2013; Kilian et al. 2017; Jones et al. 2018; Güzel et al. 2021; Yin et al. 2022). *L.sagittarioides*, however, is the first case with evidence for a putative reticulation event between ancestors of the *Lactuca* lineage and the *Notoseris-Paraprenanthes* lineage(s). Low statistical support for, and morphological discrepancies of *L.sagittarioides* with the sister groups resolved in the nrITS tree and the plastid tree, make it likely that both parental ancestors are extinct.

Taxa supposed to have evolved from ancient intergeneric reticulation events would, for consistency, be treated in a phylogeny-based classification as nothogenera. The relationships of the Lactucinae lineages tentatively classified at generic rank (Kilian et al. 2017) are, however, incompletely resolved. For any revised classification the relationships of these lineages are essential. The inferred relative frequency of ancient reticulation events in the Lactucinae show that the barriers between different lineages were rather weak at times and that may also have added to the shallow morphological differentiation between the different lineages treated as genera. In particular, the separation of *Lactuca* and *Melanoseris* is strongly questionable (Güzel et al. 2021). For the time being, creating nothogenera seems therefore premature and a classification of ancient hybridogenous lineages according to the nuclear tree the preferable interim solution, considering the essential weight of the nuclear genome or the nature of the taxon.

## ﻿Taxonomy

We treat the taxon in accordance with the findings of the nuclear ribosomal ITS phylogeny as a member of *Lactuca*.

### ﻿ Lactuca sagittarioides

Taxon classificationPlantaeAsteralesAsteraceae

C.B.Clarke, Compos. Ind.: 265. 1876.

EB11CF8F-5D53-5815-A8D0-8B760D9E5175

≡ Ixerissagittarioides (C.B.Clarke) Stebbins in J. Bot. 75: 51. 1937.
≡ Ixeridiumsagittarioides (C.B.Clarke) Pak & Kawano in Mem. Fac. Sci. Kyoto Univ., Ser. Biol. 15: 48. 1992.
≡ Mycelissagittarioides (C.B.Clarke) Sennikov in Bot. Zhurn. 82(5): 112. 1997.

#### Syntypes.

“Himalaya boreali-occidentali”, 6000’, *T. Thomson* (K); [India, Uttarakhand, Kumaon Hills] “Nynee Tal” [= Nainital], *T. Thomson* (K); [India, Himachal Pradesh, Punjab] “Dhurmsala” [= Dharamshala], *C.B. Clarke* (K); Nepal, 3.1821, *Wallich Cat. 3270* (K001118954, digit. image!; BM 000035537, digit. image!); Burma, ad Moyen, 1200’, *J. Anderson* (K).

#### Description.

Perennial rosette herb, (15–)20–65 cm tall; caudex small, often branched and plant with a two or a few rosettes. Taproot cylindric to narrowly turniplike, to c. 1 cm in diam.; lateral roots perhaps also shoot-bearing. Stem usually one per rosette, erect, branched from basal half or higher up, leafless or with few leaves in proximal portion, sparsely hairy. Rosette leaves conspicuously sagittiform and usually long-petiolate; petiole 2–22 cm, narrowly winged, margin entire or distantly sinuate-dentate; lamina triangular in outline, 2–8 × 1.5–10 cm, usually with a basal pair of acute to acuminate triangular lateral lobes and an acute triangular terminal lobe; the lateral lobes narrow or broad, sometimes much reduced to missing, directed downwards, outwards or upwards; sometimes lamina with an additional rudimentary pair of lobes above the basal one and then pentagonal; margin shallowly sinuate-dentate and often also denticulate. Stem leaves few, the lower ones similar to basal leaves but smaller and less lobed, upper leaves lanceolate to linear-lanceolate, entire, narrowed into short petiolate portion. Synflorescence paniculiform-corymbiform, with some to many capitula. Capitula with c. 12–25 florets; peduncle wiry, mostly 1–2 cm long. Involucre narrowly cylindric, 7–8 mm at anthesis to 8–10 mm at fruiting; outer phyllaries narrowly ovate to lanceolate, apex acute, outermost c. 2 mm, innermost up to 2/3 of the length of the inner (rarely longer); inner phyllaries c. 8(–10), linear-lanceolate, acute. Florets with [orange-, according to collector] yellow corolla, c. 12–14 mm; ligulae c. 6–8 mm; anther tube yellow, fertile portion c. 2.2 mm, apical appendages 0.2 mm, basal appendages 0.4–0.5 mm; style yellow. Achenes 4–4.5 mm long, slightly compressed, subfusiform with largest diameter in middle third, apically attenuate into a beak, basally less strongly attenuate into an annular carpophore; corpus with 5 main ribs, each with ±2 secondary ribs; scabrid of antrorse triangular apical projections of achene epidermis cells, brown to purplish brown; beak 0.6–1.2(–2) mm, pale. Pappus 4–6 mm, persistent, of scabrid, white bristles.

#### Variation.

Although the leaves are always distinctly sagittiform, their shape and size underlie considerable variation. The shape variation given in the description is believed to circumscribe its full extent, but we are uncertain whether the size variation is fully covered in the description. A sterile specimen from NW Himalaya (Chamba state, Kuntha Forest, Aug 1898, J.H. Lace 14C (E00360966) only including a leaf rosette may belong to *Lactucasagittarioides*; its leaves approach 40 cm in length, with a petiole of up to 30 cm, and a lamina of up to 10 × 20 cm. The beak length of the achenes usually ranges between 0.6–1.2 mm, but Gamble 23483 from NWP has a beak of c. 2 mm; its innermost outer phyllaries are unusually long, approaching the inner in length.

#### Specimens seen.

India. Uttarakhand: Kumaon, Lohba, 5500’ Apr 1848, R. Strachey & J.B. Winterbottom (K); Kumaon, Gungoli, 5300’, R. Stratchey & J.S. Winterbottom (BM 011024277, digit. image). – Uttar Pradesh, [“North western Province, Jannsar[?] District, 3000’, May 1892, J.S. Gamble (K).

Bhutan. Khine Lhakang, 6000’, 15 Apr 1949, F. Ludlow et al. 20135 (BM 000035434, digit. image)

Burma. Shan hills Matean[?] near Wankou[?], 5000’, Mar 1888, H. Collett 471 (K); Mundat, 4800’, 29 Apr 1956, F. Kingdon-Ward 22171 (BM 11024278, digit. image).

Thailand. Chiengmai, Doi Sutep, open *Quercus* forest, 1250 m, 18 Apr 1958, T. Sørensen et al. 2876 (C, digit. image)

China. Yunnan: Salween valley, 25°6'N, 98°50'E, slopes, dry grassy banks, Apr 1931 G. Forrest 29519 (E 00489230, PE); Jengyueh, 25°N, 98°36'E, 5000–7000’, hills, dry clay pasture, Aug 1924, G. Forrest 24794 (E00489233); Jengyueh, 25°N, 98°36'E, 5000–7000’, hills, open pasture, Mar 1924 G. Forrest 24004 (E00489232); Jengyueh, 25°N, 98°36'E, 7000’, hills, open stony clay pasture, Apr 1925, G. Forrest 26308 (E 00489231; K, PE); [...], S.W. grass mts, 5000’, A. Henry 12998 (K); Jingdong, San Cha Ho, 24°36'56"N, 100°42'35"E, 1600 m, 13 Mar 1940, M.G. Li 1884 (KUN); Shuangjiang, 23°28'24.6"N, 99°49'39.72"E, 1068 m, Apr 1936, C.W. Wang 72957 (KUN, PE); Xingping, Pingdian, 24°01'21"N, 101°52'20"E, 1326 m, 1 Jun 2012, Xingping survey team 5304270356 (IMDY); Menghai, Meng’e, 22°13'30"N, 100°17'49"E, 1195 m, Menghai survey team 5328220572 (IMDY); Jinghong, Caiyang River, 22°09'23.51"N, 101°11'59.28"E, 1250 m, Jinghong survey team 5328010664 (IMDY). – Guizhou: Wangmo, 25.18°N, 106.12°E, 700 m, 13 Apr 2018, *J.W. Zhang 1091* (KUN).

#### Distribution.

*Lactucasagittarioides* is distributed along the Himalayan chain from N Pakistan across NW India, Nepal, Bhutan, N Myanmar and N Thailand to SW China (for references see Kilian et al. 2009b). It is found on open, often grassy slopes, at altitudes mostly between 1500 and 2000 m, but down to 700 m in Guizhou and up to somewhat above 2000 m in Yunnan. The occurrence of the species seems altogether very scattered and it does not seem to be frequent anywhere.

## Supplementary Material

XML Treatment for Lactuca sagittarioides

## ﻿Additional information

### Author ORCIDs

Jian-Wen Zhang https://orcid.org/0000-0002-4735-168X

Norbert Kilian https://orcid.org/0000-0003-0890-4373

### Data availability

All of the data that support the findings of this study are available in the main text.

## References

[B1] Bennett L Melchers B Proppe B (2020) Curta: a general-purpose high-performance computer at ZEDAT, Freie Universität Berlin. Refubium, Dokumente FU. 10.17169/refubium-26754 [Accessed 15 March 2021]

[B2] Bentham G Hooker JD (1873) Genera plantarum, vol. 2(1). Londini [London], Lovell Reeve & Co. 10.5962/bhl.title.747

[B3] Bergsten J (2005) A review of long-branch attraction. Cladistics 21(2): 163–193. 10.1111/j.1096-0031.2005.00059.x 34892859

[B4] Bremer K (1994) Asteraceae. Cladistics and classification. Portland, Timber.

[B5] Clarke CB (1876) Compositae indicae descriptae et secus genera *Benthamii* ordinatae. Calcutta, Thacker, Spink and Company. 10.5962/bhl.title.49202

[B6] Darriba D Posada D Kozlov AM Stamatakis A Morel B Flouri T (2019) ModelTest-NG: A new and scalable tool for the selection of DNA and protein evolutionary models. Molecular Biology and Evolution 37(1): 291–294. 10.1093/molbev/msz189 PMC6984357 31432070

[B7] Güzel ME Coşkunçelebi K Kilian N Makbul S Gültepe M (2021) Phylogeny and systematics of the Lactucinae (Asteraceae) focusing on their SW Asian centre of diversity. Plant Systematics and Evolution 307(1): 1–14. 10.1007/s00606-020-01719-y

[B8] Haque MZ Godward MBE (1984) New records of the carpopodium in Compositae and its taxonomic use. Botanical Journal of the Linnean Society 89(4): 321–340. 10.1111/j.1095-8339.1984.tb02564.x

[B9] Huelsenbeck JP Larget B Alfaro ME (2004) Bayesian phylogenetic model selection using reversible jump Markov chain Monte Carlo. Molecular Biology and Evolution 21(6): 1123–1133. 10.1093/molbev/msh123 15034130

[B10] Jones KE Schilling EE Dias EF Kilian N (2018) Northern Hemisphere disjunctions in *Lactuca* (Cichorieae, Asteraceae): Independent Eurasia to North America migrations and allopolyploidization. Willdenowia 48(2): 259–284. 10.3372/wi.48.48206

[B11] Katoh K Rozewicki J Yamada KD (2017) MAFFT online service: Multiple sequence alignment, interactive sequence choice and visualization. Briefings in Bioinformatics 20(4): 1160–1166. 10.1093/bib/bbx108 PMC6781576 28968734

[B12] Kilian N Gemeinholzer B Lack HW (2009a) Tribe Cichorieae. In: Funk VA Susanna A Stuessy T Bayer R (Eds) Systematics, evolution, and biogeography of the Compositae.IAPT, Vienna, 343–383.

[B13] Kilian N Hand R von Raab-Straube E [Eds] (2009b[+]) Cichorieae Systematics Portal. http://cichorieae.e-taxonomy.net/portal/

[B14] Kilian N Sennikov A Wang Z-H Gemeinholzer B Zhang J-W (2017) Sub-Paratethyan origin and Middle to Late Miocene principal diversification of the Lactucinae (Compositae: Cichorieae) inferred from molecular phylogenetics, divergence-dating and biogeographic analysis. Taxon 66(3): 675–703. 10.12705/663.9

[B15] Kozlov AM Darriba D Flouri T Morel B Stamatakis A (2019) RAxML-NG: A fast, scalable and user-friendly tool for maximum likelihood phylogenetic inference. Bioinformatics (Oxford, England) 35(21): 4453–4455. 10.1093/bioinformatics/btz305 31070718 PMC6821337

[B16] Lee-Yaw JA Grassa CJ Joly S Andrew RL Rieseberg LH (2018) An evaluation of alternative explanations for widespread cytonuclear discordance in annual sunflowers (*Helianthus*). The New Phytologist 221(1): 515–526. 10.1111/nph.15386 30136727

[B17] Liu Y Chen Y-S Yang Q-E (2013) Generic status, circumscription, and allopolyploid origin of *Faberia* (Asteraceae: Cichorieae) as revealed by ITS and chloroplast DNA sequence data. Taxon 62(6): 1235–1247. 10.12705/626.14

[B18] Müller K (2004) PRAP— Computation of Bremer support for large data sets. Molecular Biology and Evolution 31: 780–782. 10.1016/j.ympev.2003.12.006 15062810

[B19] Müller K (2005) SeqState: Primer design and sequence statistics for phylogenetic DNA datasets. Applied Bioinformatics 4: 65–69. 10.2165/00822942-200504010-00008 16000015

[B20] Müller K Müller J Quandt D (2010) PhyDE: Phylogenetic Data Editor, version 0.9971. http://www.phyde.de/index.html

[B21] Nakamura K Chung S-W Kono Y Ho M-J Hsu T-C Peng C-I (2014) *Ixeridiumcalcicola* (Compositae), a new limestone endemic from Taiwan, with notes on its atypical basic chromosome number, phylogenetic affinities, and a limestone refugium hypothesis. PLoS ONE 9(10): e109797. 10.1371/journal.pone.0109797 PMC4190409 25295587

[B22] Pak J-H (1993) Taxonomic implications of fruit wall anatomy and karyology of Crepissect.Ixeridopsis (Compositae, Lactuceae). Korean Journal of Plant Taxonomy 23: 1–11. 10.11110/kjpt.1993.23.1.011

[B23] Pak J-H Kawano S (1990) Biosystematic studies on the genus *Ixeris* and its allied genera (Compositae-Lactuceae) I. Fruit wall anatomy and its taxonomic implications. Acta Phytotaxonomica et Geobotanica 41: 43–60.

[B24] Pak J-H Kawano S (1992) Biosystematics studies on the genus *Ixeris* and its allied genera (Compositae-Lactuceae) IV. Taxonomic treatments and nomenclature. Memoirs of the Faculty of Science Kyoto University 15: 29–61. [Series of Biology]

[B25] Ronquist F Teslenko M Van der Mark P Ayres DL Darling A Höhn S Larget B Liu L Suchard MA Huelsenbeck JP (2012) MrBayes 3.2: Efficient Bayesian phylogenetic inference and model choice across a large model space. Systematic Biology 61(3): 539–542. 10.1093/sysbio/sys029 22357727 PMC3329765

[B26] Sennikov AN (1997) Critical notes on the species of the subtribes Lactucinae and Crepidiinae (Asteraceae, Lactuceae) from Mongolia, China and Vietnam. Botanicheskii Zhurnal (St Petersburg) 82: 110–117.

[B27] Shi Z Ge X Kilian N Kirschner J Štěpánek J Sukhorukov AP Mavrodiev EV Gottschlich G (2011) Cichorieae. In: Wu ZY Raven PH Hong DY (Eds) Flora of China Volume 20–21 (Asteraceae).Beijing, Science Press; St. Louis, Missouri Botanical Garden Press, 195–353.

[B28] Simmons MP Ochoterena H (2000) Gaps as characters in sequence-based phylogenetic analyses. Systematic Biology 49(2): 369–381. 10.1093/sysbio/49.2.369 12118412

[B29] Stebbins GL (1937a) Critical notes on the genus *Ixeris*. Le Journal de Botanique 75: 43–51.

[B30] Stebbins GL (1937b) Critical notes on *Lactuca* and related genera. Le Journal de Botanique 75: 12–18.

[B31] Stebbins GL (1940) Studies in Cichorieae: *Dubyaea* and *Soroseris*. Endemics of the Sino-Himalayan region. Memoirs of the Torrey Botanical Club 19: 1–76.

[B32] Storetvedt KM (1990) The Tethys Sea and the Alpine-Himalayan orogenic belt; mega-elements in a new global tectonic system. Physics of the Earth and Planetary Interiors 62(1–2): 141–184. 10.1016/0031-9201(90)90198-7

[B33] Stöver BC Müller K (2010) TreeGraph 2: Combining and visualizing evidence from different phylogenetic analyses. BMC Bioinformatics 11(1): 7. 10.1186/1471-2105-11-7 20051126 PMC2806359

[B34] Swofford DL (2003) PAUP*: Phylogenetic Analysis Using Parsimony (*and other methods), version 4.0b 10. Sunderland, Sinauer Associates Inc. Publishers.

[B35] Wang Z-H Peng H Kilian N (2013) Molecular phylogeny of the *Lactuca* alliance (Cichorieae subtribe Lactucinae, Asteraceae) with focus on their Chinese centre of diversity detects potential events of reticulation and chloroplast capture. PLoS ONE 8(12): e82692. 10.1371/journal.pone.0082692 PMC3871690 24376566

[B36] Wang Z-H Kilian N Chen Y-P Peng H (2020) *Sinoseris* (Crepidinae, Cichorieae, Asteraceae), a new genus of three species endemic to China, one of them new to science. Willdenowia 50(1): 91–110. 10.3372/wi.50.50109

[B37] Yin Z-J Wang Z-H Kilian N Liu Y Peng H Zhao MX (2022) *Mojiangiaoreophila* (Crepidinae, Cichorieae, Asteraceae), a new species and genus from Mojiang County, SW Yunnan, China, and putative successor of the maternal *Faberia* ancestor. Plant Diversity 44(1): 83–93. 10.1016/j.pld.2021.06.007 35281119 PMC8897168

[B38] Zhang J-W Boufford DE Sun H (2011a) *Parasyncalathium* J.W. Zhang, Boufford & H. Sun (Asteraceae, Cichorieae): A new genus endemic to the Himalaya-Hengduan Mountains. Taxon 60(6): 1678–1684. 10.1002/tax.606012

[B39] Zhang JW Nie ZL Wen J Sun H (2011b) Molecular phylogeny and biogeography of three closely related genera, *Soroseris*, *Stebbinsia*, and *Syncalathium* (Asteraceae, Cichorieae), endemic to the Tibetan Plateau, SW China. Taxon 60(1): 15–26. 10.1002/tax.601003

